# Early and late effects of electroconvulsive therapy associated with different temporal lobe structures

**DOI:** 10.1038/s41398-020-01025-8

**Published:** 2020-10-13

**Authors:** Shimpei Yamasaki, Toshihiko Aso, Jun Miyata, Genichi Sugihara, Masaaki Hazama, Kiyotaka Nemoto, Yujiro Yoshihara, Yukiko Matsumoto, Tomohisa Okada, Kaori Togashi, Toshiya Murai, Hidehiko Takahashi, Taro Suwa

**Affiliations:** 1 Department of Psychiatry, Graduate School of Medicine, Kyoto University, Kyoto, Japan; 2 Laboratory for Brain Connectomics Imaging, RIKEN Center for Biosystems Dynamics Research, Kobe, Japan; 3 Human Brain Research Center, Graduate School of Medicine, Kyoto University, Kyoto, Japan; 4 Department of Psychiatry and Behavioral Sciences, Graduate School of Medical and Dental Sciences, Tokyo Medical and Dental University, Tokyo, Japan; 5 Department of Psychiatry, Faculty of Medicine, University of Tsukuba, Ibaraki, Japan; 6 Department of Diagnostic Imaging and Nuclear Medicine, Graduate School of Medicine, Kyoto University, Kyoto, Japan

**Keywords:** Biomarkers, Depression

## Abstract

Recent studies examining electroconvulsive therapy (ECT) have reported that early sessions can induce rapid antidepressant and antipsychotic effects, and the early termination of ECT was reported to increase the risk of relapse. We hypothesized that different neural mechanisms associated with the therapeutic effects of ECT may be involved in the different responses observed during the early and late periods of ECT treatment. We investigated whether these antidepressant and antipsychotic effects were associated with temporally and spatially different regional gray matter volume (GMV) changes during ECT. Fourteen patients with major depressive disorder, with or without psychotic features, underwent 3-Tesla structural magnetic resonance imaging scans before (time point [Tp] 1), after the fifth or sixth ECT session (Tp2), and after ECT completion (Tp3). We investigated the regions in which GMV changed between Tp1 and Tp2, Tp2 and Tp3, and Tp1 and Tp3 using voxel-based morphometry. In addition, we investigated the association between regional GMV changes and improvement in depressive or psychotic symptoms. GMV increase in the left superior and inferior temporal gyrus during Tp1–Tp2 was associated with improvement in psychotic symptoms (*P* < 0.025). GMV increase in the left hippocampus was associated with improvement of depressive symptoms in Tp2–Tp3 (*P* < 0.05). Our findings suggest that different temporal lobe structures are associated with early antipsychotic and late antidepressant effects of ECT.

## Introduction

Electroconvulsive therapy (ECT) is considered a rapid and reliable treatment for major depressive disorder (MDD)^1^. Courses of ECT are typically delivered over a total of 6–12 sessions, with two or three sessions per week. Symptom improvements observed during the early and late periods of ECT may be associated with different mechanisms. A rapid and strong antidepressant effect can be observed during the early period, typically before six sessions^2^, whereas the termination of ECT before attaining remission has been reported to increase the risk of relapse^3^. Several reports have indicated that antipsychotic effects also begin to emerge in the early period, as suggested by better^4^ and faster^5^ responses to ECT in patients who have MDD with psychotic features, in comparison with those without psychotic features. Separate mechanisms may produce the rapid and strong therapeutic effects observed during the early period from those related to relapse prevention during the late period of ECT.

To date, a number of magnetic resonance imaging (MRI) studies have revealed that gray matter volume (GMV) increases after ECT compared with baseline in the limbic regions, prefrontal cortex, and temporal cortex^6–14^. However, it is unclear at which point during the course of ECT this regional volume change occurs. Two previous studies reported no changes in volume before and after the first or second ECT sessions^10,14^. Therefore, volume changes may occur at or after the midpoint of the course. This possibility is in accord with the notion that therapeutic effects typically appear after the first three to six ECT sessions^2^.

It is currently unclear which regional volume changes are associated with improvement in the symptoms of MDD. Increased GMV in the hippocampus (HC) is currently the most consistent finding after ECT^15,16^. However, two meta-analyses revealed that such a volume increase was not associated with improvement in depressive symptoms^15,16^. Moreover, it is currently unclear whether an increase in GMV is associated with improvement of psychotic symptoms in MDD. Only a small number of studies have included patients who have MDD with psychotic features^11,12,14^. However, to date, no studies have assessed the differences in volume increase between patients who have MDD with and without psychotic features.

Thus, it is currently unclear whether there are differential associations between early and late therapeutic effects of ECT and regional GMV changes. To address this issue, we performed a longitudinal assessment of patients with MDD, at three time points: before, during, and after ECT. At each time point, we evaluated depressive symptoms with or without psychotic features and structural MRI images.

The aims of the study were to investigate: (i) when during the course of ECT regional GMV changes take place; (ii) whether regional volume changes in the early or late period of an ECT course are associated with depressive symptom improvement; and (iii) whether there is a difference in GMV changes between patients with and those without psychotic symptoms.

## Patients and methods

### Participants

Fourteen inpatients were recruited from the Department of Psychiatry at Kyoto University Hospital, Kyoto, Japan. All patients met the application criteria of ECT, according to American Psychological Association taskforce guidelines^17^. As for the inclusion criteria, all patients met the diagnosis of a depressive episode according to the Diagnostic and Statistical Manual of Mental Disorders, Fourth Edition, Text Revision or Fifth Edition (DSM-IV-TR or -5) and did not respond adequately to two or more different antidepressant treatments of more than 4 weeks’ duration. Exclusion criteria for all individuals were a history of severe medical conditions; current substance abuse; or a history of seizure disorder, cerebrovascular disease, or brain injury. Thirteen patients were taking one or more antidepressant medications at the time of the study: three patients were taking tricyclic antidepressants, nine were taking selective serotonin reuptake inhibitors, one was taking selective serotonin noradrenaline reuptake inhibitors, and one was taking other types of antidepressants. Seven patients exhibited psychotic features and the other seven did not exhibit psychotic features.

The study was approved by the Committee on Medical Ethics of Kyoto University and was implemented in accordance with the Declaration of Helsinki. Written informed consent was obtained from all participants.

### Electroconvulsive therapy

Bifrontotemporal ECT was administered once or twice a week using a brief-pulse square-wave ECT device (Thymatron DGX; Somatics, LLC, Lake Bluff, IL, USA). Benzodiazepines and mood stabilizers (i.e., lithium, valproate, carbamazepine, and lamotrigine) were discontinued before ECT, but antidepressants and antipsychotics were continued. Propofol (1–2 mg/kg body weight, intravenous [i.v.]) or thiopental sodium (2–4 mg/kg body weight, i.v.) were used to induce anesthesia, and succinylcholine chloride (1 mg/kg, i.v.) was used to induce muscle relaxation. The stimulus intensity for the first ECT session was determined according to the half-age stimulation strategy^18^; the subsequent intensity was adjusted based on seizure quality (monitored with motoric seizure and electroencephalogram manifestations). ECT sessions were performed until the patient reached a plateau of improvement over the previous two sessions.

### Assessment timing

We assessed clinical symptoms and acquired MRI data at three time points; before the first ECT session, after the fifth or sixth ECT session, and after completing all ECT sessions. These time points were denoted Tp1, Tp2, and Tp3, respectively.

### Clinical assessment

Depressive symptoms were evaluated using the Hamilton 17-item Depression Rating Scale (HDRS). Psychotic features were defined as the presence or absence of delusions and/or hallucinations during an episode of major depression, according to the DSM-IV-TR or DSM-5.

### Structural MRI acquisition

Each patient was scanned three times at each time point using a 3-Tesla MRI scanner with a 32-channel head coil (Skyra®; Siemens, Munich, Germany). High-resolution anatomical images were acquired in the sagittal mode using three-dimensional (3D) T1-weighted magnetization-prepared rapid acquisition of a gradient echo (MPRAGE: repetition time [TR] = 2300 ms, echo time [TE] = 2.98 ms, field of view = 232 × 256 mm, flip angle = 9°, 224 slices, matrix = 232 × 256, and final voxel size = 1 × 1 × 1 mm).

The first scan was performed within 1 week prior to the first ECT and the final scan was done within 1 week of the final ECT.

### Image preprocessing

Longitudinal brain volume changes were evaluated using voxel-based morphometry^19^. Preprocessing was performed using the Statistical Parametric Mapping software package (SPM12; The Wellcome Department of Imaging Neuroscience, London, UK), and its toolbox, the Computational Anatomy Toolbox (CAT12; http://www.neuro.uni-jena.de/cat) in MATLAB 2016b (MathWorks, Natick, MA, USA). MPRAGE data were visually checked for gross anomalies and artifacts, and reoriented to adjust image origins at the anterior commissure. Images were segmented into gray matter, white matter, and cerebrospinal fluid. The segmentation process was further extended by accounting for partial volume effects, applying adaptive maximum a posteriori estimation and using a hidden Markov random field model. Segmented gray matter images were normalized to Montreal Neurological Institute space using a template that had already been obtained with Diffeomorphic Anatomical Registration Through Exponentiated Lie algebra^20^ and the geodesic shooting algorithm^21^ in CAT12. Modulation was applied to convert the voxel values of tissue density to measures of volume. Finally, modulated gray matter images were smoothed using a Gaussian kernel of 8 mm full-width at half-maximum.

We did not use a longitudinal preprocessing pipeline implemented in CAT12 because this pipeline required all time-point data for calculation; that is, the Tp3 image was required to calculate the difference between Tp1 and Tp2, which might reduce sensitivity.

### Statistical analyses

#### 1. Demographic data and clinical characteristics

Demographic and behavioral data were analyzed using *t*-tests or chi-square tests, as appropriate. Changes in HDRS scores were evaluated using repeated measures one-way analysis of variance (ANOVA) and paired *t*-tests for post hoc testing. Additionally, HDRS score changes between Tp1–Tp2 and Tp2–Tp3 were compared using unpaired *t*-tests, to estimate differences between early and late antidepressant effects of ECT. Results were considered statistically significant at *P* < 0.05. All analyses were conducted using IBM SPSS 22 (IBM Corp., Armonk, NY, USA).

#### 2. Neuroimaging analyses

(i) Regional GMV change during the course of ECT
  To investigate regional GMV changes during the course of ECT, one-way repeated measures ANOVA, including all 14 patients, was performed, using SPM12, with F-contrasts testing of Tp1 versus Tp2, Tp2 versus Tp3, and Tp1 versus Tp3. Statistical significance was tested with a cluster-forming threshold (CFT) of *P* = 0.001 and cluster-wise family-wise error (FWE) rate of *P* < 0.05. Age, sex, total intracranial volume, and psychotic symptoms were included as covariates. Post hoc analyses were performed as paired Student’s *t*-tests comparing Tp1 versus Tp2, Tp1 versus Tp3, and Tp2 versus Tp3. The paired Student’s *t*-tests were performed using a CFT of *P* = 0.001 and a cluster-wise FWE of *P* < 0.05. Then, the resulting images were binarized to create inclusion masks, and these masks were applied to the ANOVA, described above. Cohen’s *d* effect size^21^ of significant volume changes was calculated.
  For the clusters detected in analysis (i), two subsequent region-of-interest (ROI) analyses were performed.
  *Subsequent ROI analysis (i)-a*
  To estimate the association between relative volume changes between Tp1 and Tp3 and the total number of ECT sessions, a simple regression was performed, as recent mega-studies have reported a significant positive slope between volume changes and the total number of ECT sessions^22,23^. Voxel values were extracted from significant clusters that were identified in the whole-brain analysis (i), and the relative volume changes at Tp2 and Tp3 were calculated as the ratio between the voxel values at Tp2 and Tp3, respectively, and the voxel value at Tp1. Relative volume changes were independent variables.
  *Subsequent ROI analysis (i)-b*
  To estimate the association between relative volume changes in the cluster and changes in HDRS scores, the relative volume changes were calculated, as described for ROI analysis (i)-a, and the changes in HDRS scores were calculated as a percentage of the change in HDRS scores between two time points. Then, simple regression analyses were performed, using the change in the HDRS score as the explanatory variable.
(ii) Association between regional GMV and changes in depressive symptom severity
  For periods in which we found significant changes in HDRS scores, we examined the regions in which volume changes were associated with changes in depressive symptoms in whole-brain analysis on SPM12, for all 14 patients. First, subtraction images between Tp1 and Tp2 and between Tp2 and Tp3 were calculated using the ImCalc function of SPM12. Second, changes in HDRS scores between Tp1 and Tp2 and between Tp2 and Tp3 were calculated as subsequent ROI analysis (i)-a. Finally, we performed multiple regression analyses in SPM12, using subtraction images as the dependent variables and HDRS change, presence/absence of psychotic features, age, sex, and total intracranial volume as the independent variables. Threshold-free cluster enhancement (TFCE) was used for multiple comparison corrections, using the SPM12 TFCE Toolbox^24^. A statistical significance threshold of *P* < 0.05. Because the TFCE toolbox does not support the ANOVA design, we did not use TFCE in (i).
(iii) Association between regional GMV changes and the presence/absence of psychotic symptoms

As in (ii), for periods in which psychotic symptoms improved, we examined the association between changes in regional GMV and changes in psychotic symptoms. We applied a two-sample *t*-test in depressive patients with/without psychotic symptoms (*N* = 7 in each group) using subtraction images between Tp1–Tp2 and between Tp2–Tp3. TFCE was used for multiple comparison corrections. A statistical significance threshold of *P* < 0.05 was divided by the number of comparisons. Age, sex, and total intracranial volume were included as covariates.

## Results

### 1. Demographic data and clinical characteristics

Demographic data are summarized in Table 1. At the end of treatment, nine patients matched the clinical response criteria (HDRS score rate of decrease > 50%) and five patients exhibited remission (HDRS score < 8). HDRS scores of all patients decreased significantly between Tp1 and Tp2 and between Tp2 and Tp3 (Tp1–Tp2, *P* < 0.001; Tp2–Tp3, *P* < 0.01; Tp1–Tp3, *P* < 0.0001). Thus, we included both periods (Tp1–Tp2 and Tp2–Tp3) in the analysis in (ii). The changes in HDRS score did not differ between Tp1–Tp2 and Tp2–Tp3 (*P* = 0.23).

**Table 1:** Patients’ demographic and clinical characteristics.

	Total patients with MDD		With psychotic features		Without psychotic features		
Demographic and clinical variables	(*N* = 14)		(*N* = 7)		(*N* = 7)		*P* value
Mean age, years (SD)	60.9	(17.2)	71.3	(8.4)	49.4	(17.9)	<0.05^a^
Sex (female), *n* (%)	6	(41.7)	3	(41.7)	3	(41.7)	1^b^
Mean duration of episode, weeks (SD)	61.7	(45.4)	51.1	(52.6)	73.3	(34.8)	0.36^a^
Mean age at onset, years (SD)	49.8	(18.6)	63.4	(17.6)	42	(13.7)	<0.05^a^
Previous ECT, *n* (%)	1	(7.1)	1	(16.7)	0	(0.0)	0.30^b^
Mean drug dose (SD)							
Antidepressants (mg, imipramine equivalents)	159.8	(96.7)	151.8	(98.8)	167.9	(101.8)	0.77^a^
Antipsychotics (mg, chlorpromazine equivalents)	66.2	(57.5)	82.1	(68.8)	50.2	(42.7)	0.31^a^
Presence of psychotic symptoms at Tp1, *n* (%)	–		7	(100.0)	–		
Presence of psychotic symptoms at Tp2, *n* (%)	–		0	(0.0)	–		
Presence of psychotic symptoms at Tp3, *n* (%)	–		0	(0.0)	–		
Mean HDRS score at Tp1 (SD)	26.6	(6.7)	30.7	(4.9)	22.4	(5.7)	<0.05^a^
Mean HDRS score at Tp2 (SD)	16.6	(6.8)	16.7	(5.2)	16.6	(8.6)	0.97^b^
Mean HDRS score at Tp3 (SD)	10.1	(6.3)	8.3	(5.5)	12	(6.9)	0.29^a^
Mean MMSE score at Tp1 (SD)	22.6	(5.9)	20.7	(4.8)	24.4	(6.6)	0.25^a^
Mean MMSE score at Tp2 (SD)	24.9	(4.6)	22.6	(5.6)	27.3	(1.6)	0.05^a^
Mean MMSE score at Tp3 (SD)	27.6	(2.2)	26.4	(2.6)	28.7	(1.0)	<0.05^a^
Responders, *n* (%)	9	(64.3)	6	(85.7)	3	(42.9)	0.094^b^
Mean number of ECT sessions (SD)	10.4	(2.6)	9.9	(3.0)	10.9	(2.3)	0.49^a^

*MDD* major depressive disorder, *SD* standard deviation, *HDRS* Hamilton Depression Rating Scale, *MMSE* Mini-mental state examination, *ECT* electroconvulsive therapy, Mini-mental state examination

^a^*T*-test for patients with and without psychotic symptoms.

^b^Chi-square test for patients with and without psychotic symptoms.

In accord with previous studies^5^, age, age at onset, and HDRS score at Tp1 were significantly higher in patients with psychotic depression compared with those who had non-psychotic depression.

Because psychotic symptoms were remitted by Tp2 in all patients with psychotic depression, we limited the analysis in (iii) to the period between Tp1 and Tp2.

### 2. Neuroimaging analyses

#### (i) Regional GMV changes during the course of ECT

**Fig. 1:**
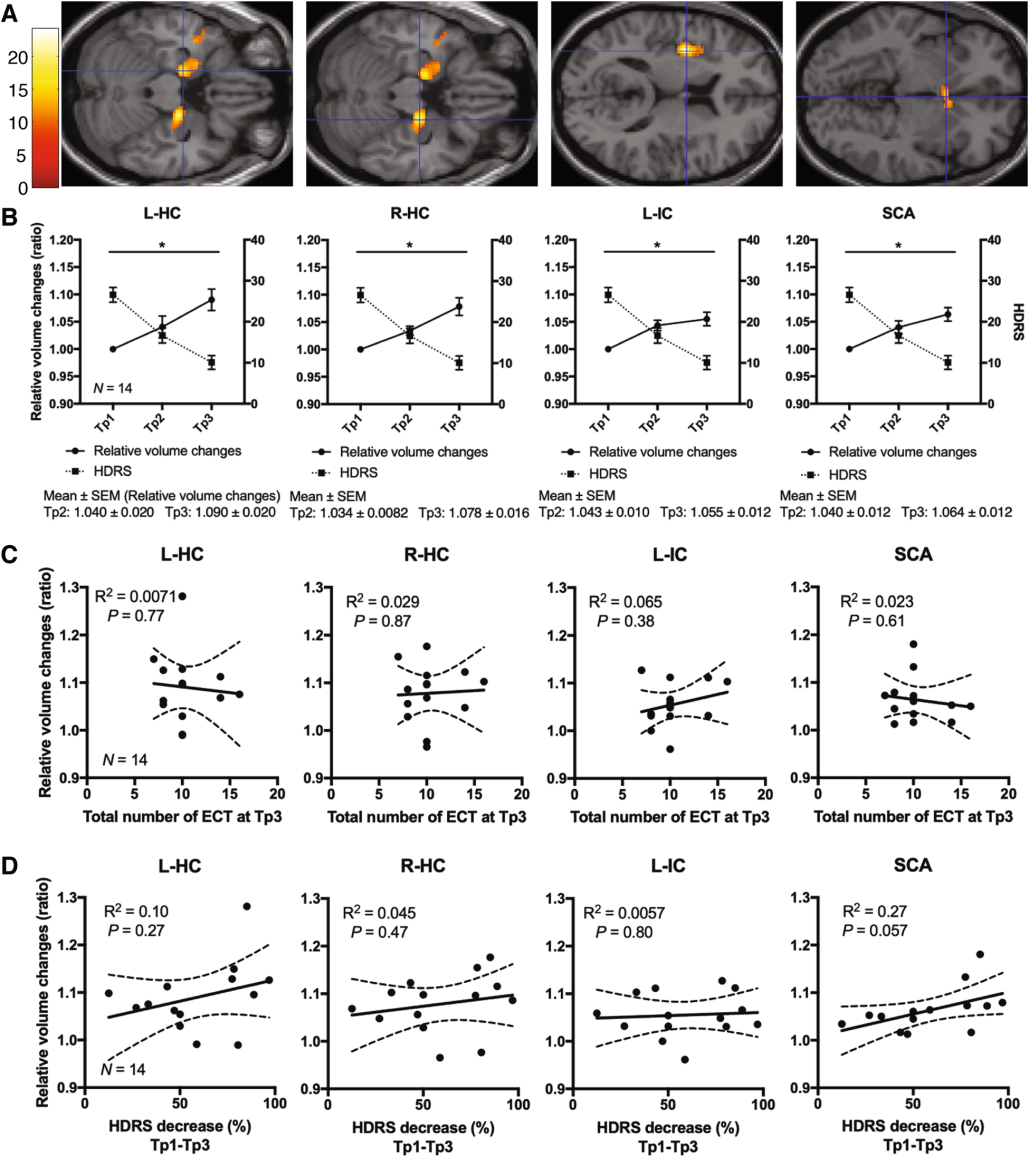
Changes in regional GMV during the course of ECT. **a** One-way ANCOVA and post hoc analysis (*N* = 14, both with and without psychotic features) revealed those brain regions in which volume changed during the course of ECT on SPM12. Cluster-forming threshold at *P* = 0.001 and family-wise error (FWE) cluster threshold at *P* = 0.05. **b** Regional GMV (*N* = 14) increased between Tp1 and Tp3 in the L-HC, R-HC, L-IC, and SCA. Solid (left *Y*-axis) and broken lines (right *Y*-axis) indicate relative volume changes compared with volume at Tp1 and HDRS scores, respectively. Error bars indicate standard error of the mean (SEM). **c** Simple regression analyses (*N* = 14) showed no significant associations between total number of ECT sessions at Tp3 and each regional volume changes. Solid and broken lines indicate regression lines and 95% confidence intervals, respectively. *R*^2^ means coefficient of determination. **d** Simple regression analyses (*N* = 14) showed no significant associations between HDRS decrease and each regional volume changes. Solid and broken lines indicate regression lines and 95% confidence intervals, respectively. *R*^2^ means coefficient of determination. ANCOVA analysis of covariance, Tp time point, ECT electroconvulsive therapy, GMV gray matter volume, L-HC left hippocampus, R-HC right hippocampus, L-IC left insular cortex, SCA subcallosal cingulate cortex.

One-way ANCOVA (*N* = 14) showed significant GMV changes during the ECT course in the left hippocampus (L-HC), right hippocampus (R-HC), left insular cortex (L-IC), and subcallosal cingulate cortex (SCA) (CFT of *P* = 0.001 and cluster-wise FWE of *P* < 0.05) (Fig. 1a). Post hoc analysis revealed significant increases in GMV between Tp1 and Tp3 in the L-HC, R-HC, L-IC, and SCA (CFT of *P* = 0.001 and cluster-wise FWE of *P* < 0.05) (Fig. 1b). The effect size of these regional volume changes between Tp1 and Tp2 and between Tp2 and Tp3 were 0.60 and 0.83, respectively, in the L-HC, 1.04 and 0.83 in the R-HC, 1.03 and 0.61 in the L-IC, and 1.26 and 0.26 in the SCA. There were no significant decreases in GMV.

*Subsequent ROI analysis (i)-a*

A simple linear regression was performed to estimate volume changes between Tp1 and Tp3, based on the total number of ECT sessions. No significant regression equation was found [L-HC: *F*(1, 12) = 0.086, *P* = 0.77, with an *R*^2^ of 0.0071; R-HC: *F*(1, 12) = 0.029, *P* = 0.87, with an *R*^2^ of 0.002, L-IC: *F*(1, 12) = 0.84, *P* = 0.38, with an *R*^2^ of 0.065; SCA: *F*(1, 12) = 0.28, *P* = 0.61, with an *R*^2^ of 0.023; Fig. 1c].

*Subsequent ROI analysis (i)-b*

A simple linear regression was performed to estimate volume changes based on decreases in HDRS. No significant regression equation was found [L-HC: *F*(1, 12) = 1.35, *P* = 0.27, with an *R*^2^ of 0.10; R-HC: *F*(1, 12) = 0.56, *P* = 0.47, with an *R*^2^ of 0.045; L-IC: *F*(1, 12) = 0.069, *P* = 080, with an *R*^2^ of 0.0057; SCA: *F*(1, 12) = 4.42, *P* = 0.057, with an *R*^2^ of 0.27; Fig. 1d].

#### (ii) Association between changes in regional GMV and depressive symptom severity

**Fig. 2:**
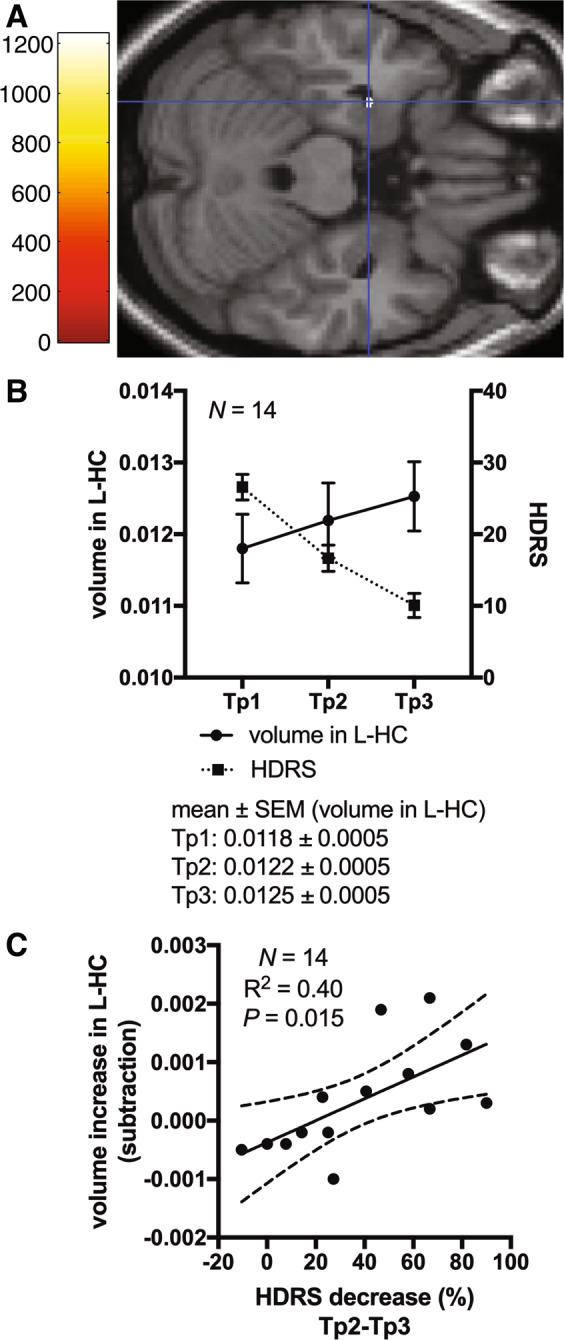
Association between changes in regional GMV and depressive symptom severity. **a** Multiple regression analysis on SPM12 (*N* = 14, both with and without psychotic features) revealed volume increase in which the cluster was located in the L-HC was dependent of a decrease in HDRS scores between Tp2 and Tp3. The statistical threshold was *P* = 0.05, TFCE. Covariates of no interest were age, sex, psychotic features, and total intracranial volume. **b** Plot of volume of the significant cluster located in L-HC, which was found in analysis ii), at each time point (*N* = 14). Solid (left *Y*-axis) and broken line (right *Y*-axis) indicates volume in the L-HC and HDRS scores, respectively. Error bars indicate standard error of the mean (SEM). **c** Plot of regression line of the volume changes in the L-HC cluster between Tp2 and Tp3 with respect to the decrease in HDRS score (*N* = 14). Solid and broken lines indicate regression lines and 95% confidence intervals, respectively. *R*^2^ means coefficient of determination. HDRS Hamilton Depression Rating Scale, TFCE threshold-free cluster enhancement, L-HC left hippocampus, SEM standard error of the mean.

Decreases in HDRS scores, between Tp2 and Tp3, were significantly associated with increases in GMV, in the cluster located in the L-HC (FWE of *P* < 0.05; Fig. 2a–c).

#### (iii) Association between changes in regional GMV and presence/absence of psychotic symptoms

**Fig. 3:**
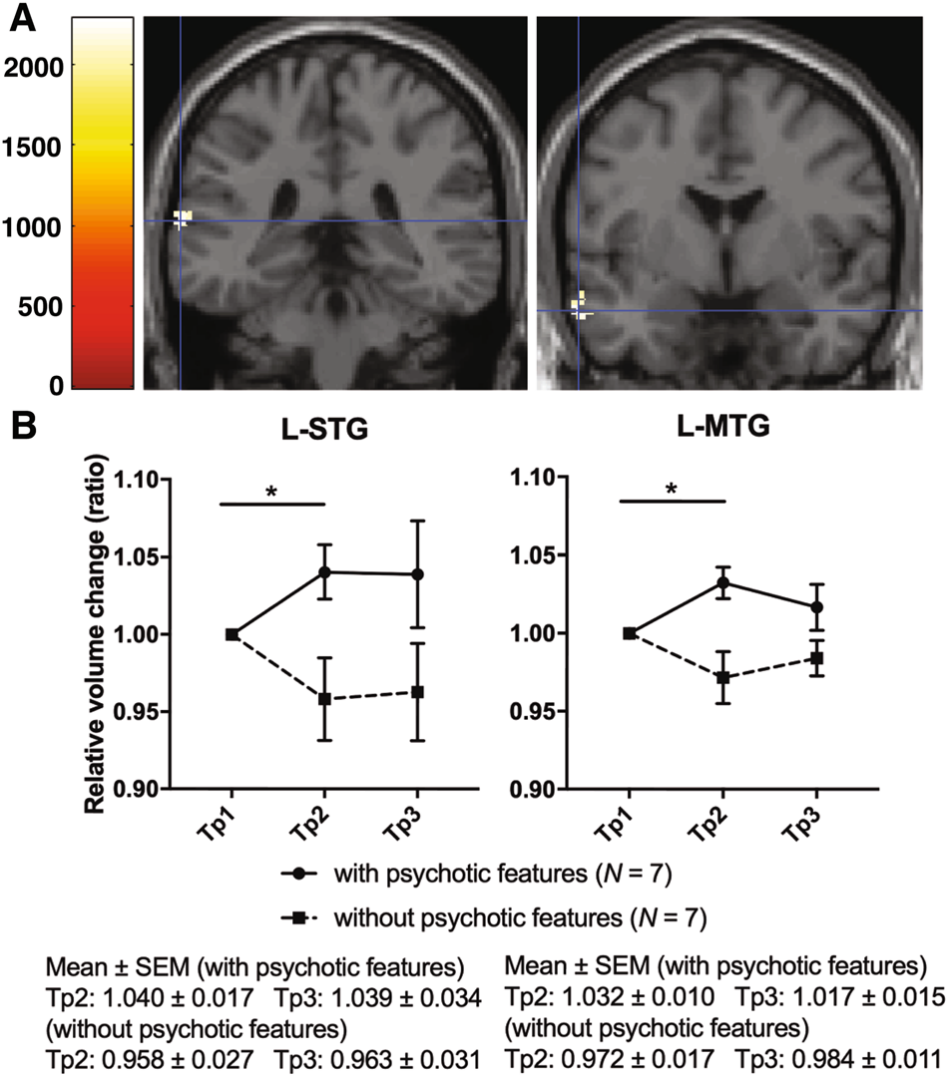
Association between changes in regional GMV and presence/absence of psychotic symptoms. **a** L-STG/MTG volume increase was observed in patients who had depression with psychotic features (*N* = 7) in comparison with those (*N* = 7) without psychotic features between Tp1 and Tp2. The statistical threshold was *P* = 0.05/2, TFCE and number of contrasts. Covariates of no interest were age, sex, and total intracranial volume. **b** Plot of the L-STG/MTG volume change at each time point in patients who had depression with (solid line) and without (broken line) psychotic features. Error bars indicate standard error of the mean (SEM). Tp time point, L-STG/MTG left superior and middle temporal gyrus, TFCE threshold-free cluster enhancement, SEM standard error of the mean.

We found significant differences in two regions between the two patient groups (those with/without psychotic symptoms, *N* = 7 in each group) between Tp1 and Tp2, in which psychotic symptoms were improved, indicating an increase in volume of the left superior and middle temporal gyrus (L-STG/MTG) in patients with psychotic features as compared with those who did not have psychotic features (*P* < 0.025, corrected for two contrasts) (Fig. 3).

## Discussion

### Summary

To our knowledge, this is the first study to demonstrate that different periods during the ECT course are associated with increases in GMV in different regions, as well as being associated with different therapeutic effects. The present study revealed four main findings:

1. Early and late periods of ECT had different therapeutic effects.
2. GMV increases were found in several regions, including the bilateral HC, at the end.
3. The GMV increase in the L-HC was associated with a decrease in depression severity during the late period of the ECT course.
4. In comparison with patients who had non-psychotic depression, patients with psychotic depression exhibited GMV increases in the L-STG/MTG during the early period of ECT.

### Differences between early and late effects of ECT

We found that the early period of ECT was associated with both antidepressant and antipsychotic effects whereas the late period was associated with antidepressant effects only. These results are compatible with those of previous studies reporting that antidepressant and antipsychotic effects begin to emerge during the early period of ECT^2,4,5^. However, we did not observe significant differences in the antidepressant effects of ECT between the early and late periods.

### Regions with longitudinal volume change

We found an increase in GMV in the bilateral HC, L-IC, and SCA after the entire ECT course (Fig. 1a), which was consistent with previous ECT studies^6–14^. We did not find a GMV increase at Tp2 as compared with Tp1, but the effect sizes of regional volume changes between Tp1 and Tp2 and Tp2 and Tp3 were large^21^. Thus, regional GMV increased gradually during the entire course of ECT, resulting in the largest regional volume at the end of the ECT course (Fig. 1b). The results are consistent with a previous study showing that no increase in volume was exhibited after a single ECT session^14^.

Two mega-studies showed a significant correlation between volume changes and the total number of ECT sessions, which suggested that more sessions resulted in increased volumes^22,23^. However, we did not find such a correlation, in this study (Fig. 1c). This difference in results may be explained by the protocol in our study, in which those subjects who responded well to ECT received fewer sessions, whereas those who responded poorly received more sessions. The volume change per session may be larger among the responsive group and smaller for the group with poor response. The inclusion of patients with different responses to treatment in our study may have led to inconsistent results. Thus, we calculated the correlation between the relative volume change per session and the total number of ECT sessions, using a simple regression analysis. The dependent variable was the per-session volume change, and the explanatory variable was the number of ECT sessions. We found no significant correlation between these two variables, but the slopes were generally negative (Supplementary Fig. 1). This result may explain the lack of significant association between volume changes and the number of ECT sessions observed in our study, together with the relatively small sample size. When assessing associations between volume changes and the number of ECT sessions, treatment responsiveness should be considered.

The microstructural mechanisms underlying our findings are beyond the scope of the current study. However, several previous animal studies have reported that electroconvulsive shock increased the number of newborn neurons in the dentate gyrus of rodents in proportion to the number of the sessions^25,26^; a similar mechanism may underlie the increase in bilateral HC volume after ECT.

### Association between depressive symptom change and the HC

Subsequent ROI analysis (i)-b identified no significant associations between the bilateral HC and HDRS increases between Tp1 and Tp3 (Fig. 1d), but our whole-brain analyses found an association between a decrease in depressive symptoms and an increase in L-HC volume in the late period of ECT (Fig. 2a, c). A previous whole-brain study showed that similar associations were found before ECT and after the ninth session of ECT^14^. Both results indicated that volume increases in the L-HC can mediate the antidepressant effects associated with middle or late course ECT, after the L-HC volume was significantly enlarged. Changes in total HC may not be necessarily be associated with the antidepressant effects of ECT. ECT increases large regions of the medial temporal lobe, including the majority of the L-HC (Fig. 1a), which was partially associated with the antidepressant effects of ECT (Fig. 2a). Thus, we should focus on the effects associated with HC subregions.

Our study design, using three time points, enabled us to detect this symptom–structure association. Recent MRI studies in humans have revealed that a volume increase in the dentate gyrus after ECT was associated with improvement in depression severity^27,28^.

In addition, animal model studies have reported that neurogenesis in the dentate gyrus is necessary to induce antidepressant effects^29–31^. Taken together, these findings suggest that ECT may exert antidepressant effects via neurogenesis in the HC, particularly the dentate gyrus.

Several studies have reported that the left hemisphere or left hippocampus may be involved in the cognitive side effects associated with ECT. Bilateral electrode positioning results in greater volume increases in the L-HC and increased cognitive side effects than right unilateral electrode positioning^22,32^. One study reported that ECT-induced cognitive impairment was associated with a volume increase in the HC^33^, whereas another study, which focused on the L-HC, did not find any such association^34^. Here, we performed two additional analyses to determine whether such an association existed. We investigated the association between changes in cognitive function, as assessed by the MMSE, and volume changes in the L-HC that were identified as significant in the following two analyzed results: (i) pre- and post-ECT comparisons (Fig. 1a) and (ii) regression analysis with improvements in depressive symptoms (Fig. 2a). However, these additional analyses showed no significant results (Supplementary Fig. 2).

### Improvement in psychotic symptoms and the superior and middle temporal gyrus

The current results revealed that remission of psychotic symptoms in the early period of ECT was associated with GMV increases in the STG and MTG (Fig. 3). The current findings may also explain the inconsistency among previous reports regarding STG/MTG volume changes induced by ECT^10,13^. Two studies that included participants with a high proportion (53% and 26%) of psychotic depression reported GMV increases in the STG/MTG, respectively^11,12^.

Previous studies reported reduced GMV in the STG/MTG of depressive patients with psychotic features^35–37^. In particular, reduced left STG/MTG GMV has been correlated with the increased severity of auditory hallucinations^33,38^. The current results showed that the GMV in the left STG/MTG could be normalized by ECT in the group with psychotic features during the early period. This result was in line with the clinical reports that patients with psychotic depression are more likely to respond to ECT earlier than patients without psychotic depression^4,5^. In contrast, the non-psychotic group experienced a reduction in STG/MTG lobe volume during the early period, although the reasons for this change are unclear because ECT increases the volumes of a wide range of regions^23^. However, volume reduction in the temporal lobe, which could be associated with depression^39^, may precede volume increases due to ECT because volume increases require a sufficient number of ECT sessions. Differences in the GMV response to ECT were not observed in any regions other than the STG/MTG. Volume increases associated with ECT may be specific to the left STG/MTG, which have also been associated with psychotic symptoms. Our hypothesis suggests that the early antipsychotic effects of ECT were associated with the increased GMV observed in the left STG/MTG, and that the subtype of depression characterized by psychotic features could have a structural brain underpinning. The association between ECT and psychotic symptoms requires further study.

### Limitations

The current study involved several limitations that should be considered. First, given the small number of patients, the results of the current study should be interpreted cautiously. Second, we were unable to recruit controls (i.e., patients who did not receive ECT and healthy subjects) with whom to compare changes in regional GMV during the observation period. Third, nearly all patients in the current study were taking medication; thus, we cannot exclude the possibility of medication effects. Fourth, we did not have a measure of the microstructural change induced by ECT. Advanced methods, such as ultra-high field MRI, myelin maps, neurite orientation dispersion, and density imaging would be useful to elucidate the microstructural basis of ECT in depression.

### Conclusion

Using a study design with assessment at three time points, we revealed that the HC volume increased slowly during the course of ECT. In addition, we found that the early antipsychotic and late antidepressant effects of ECT were associated with volume increases in the STG/MTG and HC, respectively. Thus, ECT may exert antidepressant effects through its effect on the HC. Future studies should investigate the mechanisms underlying the effects of ECT using advanced imaging methods that are sensitive to microstructural changes.

## Supplementary information

Supplemental material
