# Supplementary material for: Early and late effects of electroconvulsive therapy associated with different temporal lobe structures
Source: Transl Psychiatry. 2020 Oct 13;10:344. doi: 10.1038/s41398-020-01025-8 (PMC7553938; doi:10.1038/s41398-020-01025-8)
Supplement: Supplementary file 1 — Supplemental material [file 41398_2020_1025_MOESM1_ESM.docx]

**Supplementary Materials**

**Supplementary Figure 1.** Association between the relative volume change per ECT session and the total number of ECT sessions.


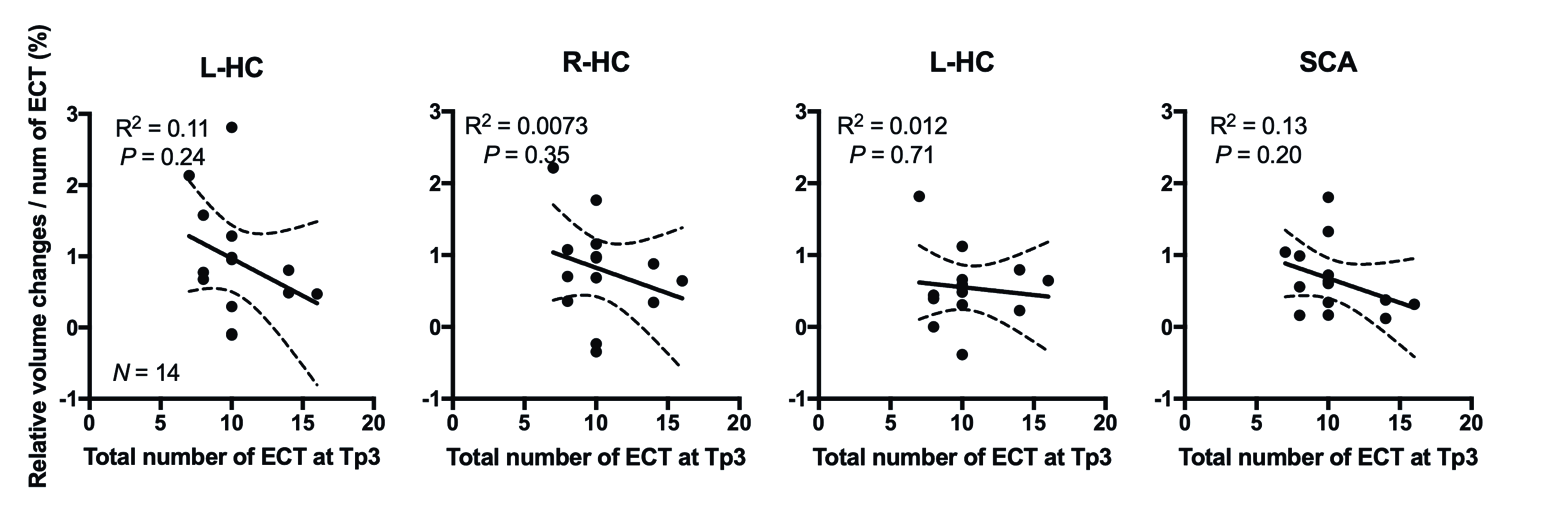


Plots, showing the regression lines for the relative volume change per ECT session against the total number of ECT sessions (*N* = 14). The solid and broken lines indicate the regression lines and 95% confidence intervals, respectively. R^2^ values indicate the coefficients of determination.

Tp, time point; ECT, electroconvulsive therapy; L-HC, left hippocampus; R-HC, right hippocampus; L-IC, left insular cortex; SCA, subcallosal cingulate cortex.

**Supplementary Figure 2.** Association between MMSE changes and volume increases in the L-HC.


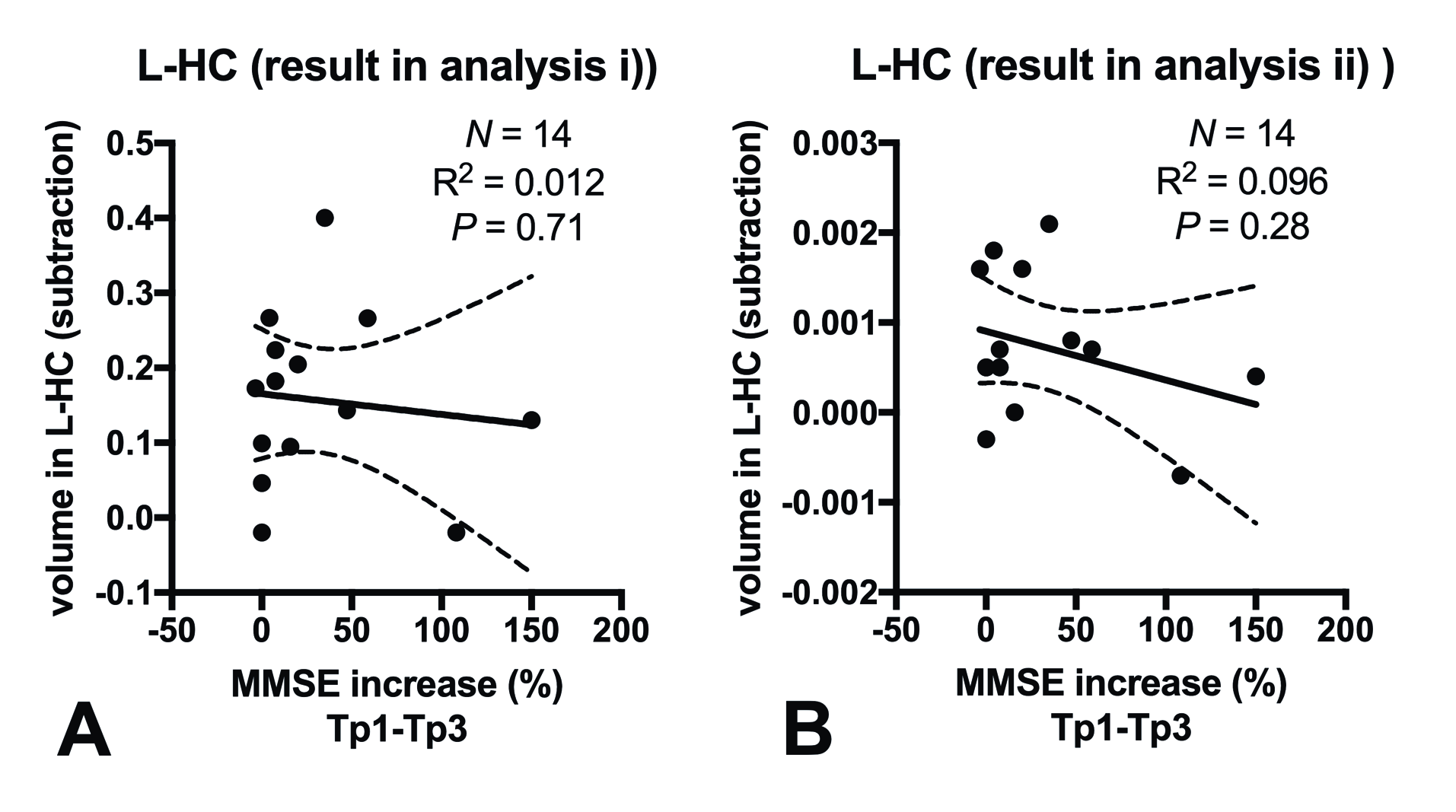


(A) Plot showing the regression line for the volume changes in the L-HC cluster [found in analysis i)] between Tp1 and Tp3, relative to the increase in MMSE (*N* = 14).

(B) Plot showing the regression line for the volume changes in the L-HC cluster [found in analysis ii)] between Tp1 and Tp3, relative to the increase in MMSE (*N* = 14).

Solid and broken lines indicate regression lines and 95% confidence intervals, respectively. R-squared indicates the coefficient of determination.

L-HC, left hippocampus; MMSE, Mini-mental state examination
